# Increased survival of patients aged 0‐29 years with osteosarcoma: A period analysis, 1984‐2013

**DOI:** 10.1002/cam4.1659

**Published:** 2018-07-10

**Authors:** Jinna Wu, Huanhuan Sun, Jie Li, Yuanqing Guo, Kuibo Zhang, Chuandong Lang, Changye Zou, Haiqing Ma

**Affiliations:** ^1^ Department of Oncology The Fifth Affiliated Hospital of Sun Yat‐sen University Zhuhai Guangdong China; ^2^ Department of Breast and Thyroid Surgery The First Affiliated Hospital of Sun Yat‐sen University Guangzhou Guangdong China; ^3^ Department of Spinal Surgery The Fifth Affiliated Hospital of Sun Yat‐sen University Zhuhai Guangdong China; ^4^ Department of Spinal Surgery The First Affiliated Hospital of Sun Yat‐sen University Guangzhou Guangdong China; ^5^ Department of Orthopedic Oncology The First Affiliated Hospital of Sun Yat‐sen University Guangzhou Guangdong China

**Keywords:** incidence, osteosarcoma, period analysis, relative survival, sex, socioeconomic status

## Abstract

**Purpose:**

Osteosarcoma is the most common primary malignancy of bone, and typically occurs among children and adolescence. This study aims to evaluate treatment outcomes among children, adolescents and young adults with osteosarcoma over the three decades by the changes in the long‐term relative survival.

**Methods:**

Osteosarcoma incidence and relative survival data from Surveillance, Epidemiology, and End Results (SEER) registries during 1984‐2013 were analyzed. The survival differences over three decades, age, sex, race, and socioeconomic status (SES) were assessed by comparing Kaplan‐Meier curves.

**Results:**

The overall incidence of osteosarcoma kept relatively stable with 0.4 per 100 000 in the three decades with the peak incidence occurring in the aged 10‐19 group. The 10‐year relative survival rate (RSR) increased from 57.7% to 61.0% in the three decades, with the greatest increase in the aged 0‐9 group from 48.2% to 65.7%. The 10‐year RSR improved from 54.1% to 61.5% in males, and from 62.4% to 63.0% in females, respectively, in the three decades. Furthermore, survival dramatically improved from 30% to 60% in the high‐poverty group over the three decades.

**Conclusion:**

This study demonstrated that the overall incidence of osteosarcoma remained stable, with an improvement in survival in the three decades. The improved survival was greater in males than in females in the three decades. Furthermore, the survival significantly increased in high‐poverty group, which was attributed to increasing improved health care system and patients with low finance can also have access to receiving effective and consistent treatment without distinction.

## 1 INTRODUCTION

Osteosarcoma, the most common primary malignancy of bone, typically occurs among children and adolescence.^1^ For young patients, it can occur in any bone with the major sites in long bone, especially in the distal femur, proximal tibia, and proximal humerus.^2^, ^3^ It was reported approximately 900 new cases of osteosarcoma diagnosed every year in the United States, accounting for less than 0.2% of all cancers, but the disease has a highly invasive and distant metastatic potential with highly lethal.^4^ With advance in treatments, such as surgical resection, chemotherapy, and radiation therapy, the overall survival of patients with osteosarcoma has improved in the past few decades.^5^, ^6^, ^7^, ^8^ We sought to determine whether the trend in increasing survival had proved among children, adolescents, and young adults over the past time.

To evaluate the treatment outcomes among children, adolescents, and young adults with osteosarcoma over the three decades, the survival data was accessed from the Surveillance, Epidemiology, and End Results (SEER) database between 1984 and 2013. In the study, period analysis was used to examine the changes in the long‐term relative survival among children, adolescents, and young adults associated with osteosarcoma. Additionally, osteosarcoma patients were stratified by age, sex, race, and socioeconomic status (SES) to compare survival disparities.

## 2 METHODS

All data of patients with osteosarcoma during 1984‐2013 were obtained from the SEER database of the National Cancer Institute. SEER*Stat version 8.3.4 was used to calculated the incidence and survival data, which collected from the original nine SEER sites and 18 SEER sites, respectively.

All osteosarcoma cases inclusion criteria were based on the International Classification of Childhood Cancers (ICCC)^9^ and/or the International Classification of Disease for Oncology (ICD‐O‐3),^10^ and the anatomic site codes and all histology subtypes were classified as described by ICD‐O‐3 codes (C40.0‐C41.9) and pathology (ICD‐O‐3 codes 9180‐9186, 9192‐9194).^11^

For this study, the incidence and relative survival data from 1984 to 2013 were analyzed after being divided by three decades. Additionally, osteosarcoma patients were stratified by different age, sex, SES and race (White, Black, and Other). The patients younger than age 30 years at primary diagnosis of osteosarcoma (age groups: 0‐9, 10‐19, and 20‐29 years) were included. The county poverty rate was used to difine area SES, which is the percentage of persons in the county living below the national poverty threshold in Census 2000.^12^ According to the National Cancer Institute monograph, the county poverty rates were divided into three levels using the same cut points: <10% (low‐poverty areas), 10%‐19.99% (medium‐poverty areas), and ≥20% (high‐poverty areas).^13^, ^14^ When analyzing the incidence, the medium‐ and high‐poverty were redefined as “med‐high‐poverty.” Osteosarcoma cases diagnosed by autopsy or reported only on a death certificate were excluded.

Incidence was expressed per 100 000 population, and the age‐adjusted rates were calculated with the 2000 U.S. standard population. Kaplan‐Meier survival analysis was used to compare the survival difference with *P* value <0.05, which was considered statistically significant. For defining age, sex, race, and SES involved in this study, which were the independent risk factors, we assessed these variables via Cox regression analysis using the STATA software (version 12.0; Stata Corp, College Station, TX). Risk factors that were served as potential importance in univariate analysis were included in the multivariate analysis. A two‐tailed *P* value <0.05 was believed statistically significant.

## 3 RESULTS

### 3.1 Incidence of osteosarcoma at nine original SEER sites

**Figure 1:**
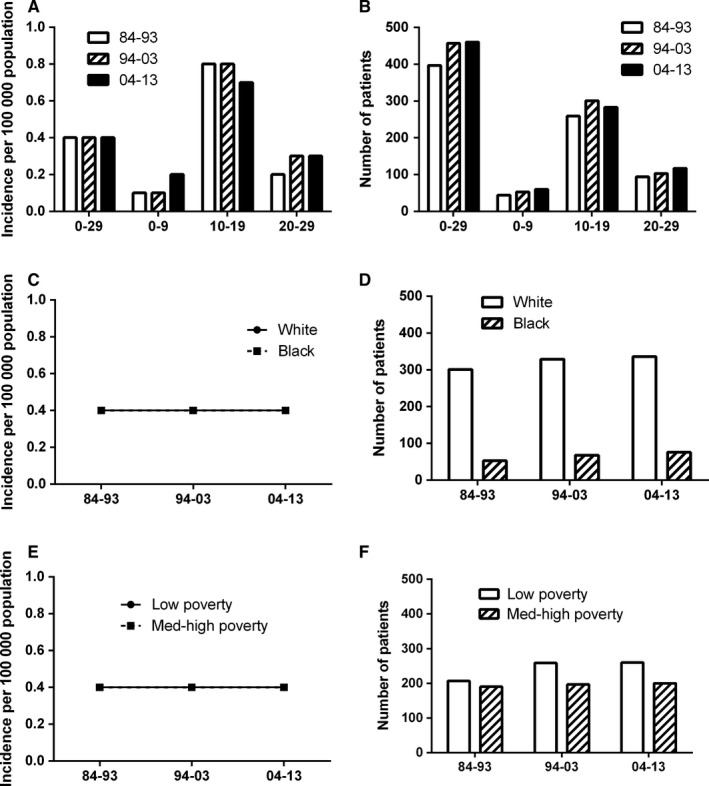
Summary incidences of patients diagnosed with having osteosarcoma between 1984 and 2013 at the original nine SEER sites. Incidence (A), and number (B) of osteosarcoma cases are shown by age group (total and age 0‐9, 10‐19, and 20‐29 years) and calendar period. Incidence (C, E) and number (D, F) of osteosarcoma cases are grouped by race and SES, respectively

Total 1314 patients aged 0‐29 years were diagnosed with osteosarcoma during 1984‐2013, and no previous cancer diagnoses were identified from the data for the nine original SEER sites. The number of osteosarcoma patients increased by 16.1% over the three decades (1984‐1993, 1994‐2003, and 2004‐2013), from 397 to 457 to 460, respectively; and it was strongly pronounced increase by 50% for aged 0‐9 group: from 40 to 53 to 60 (Figure 1, TableS1). More males than females aged 0‐29 years were diagnosed with osteosarcoma; the absolute number of all patients with osteosarcoma, respectively, increased from 227 to 256 in males, and from 170 to 204 in females over the past three decades.

The overall incidence of osteosarcoma kept relatively stable with 0.4 per 100 000 in the three decades. Although the peak incidence occurred in the aged 10‐19 group, it declined by 12.5% from 0.8 to 0.7 per 100 000 in the three decades. However, the incidence increased from 0.1 to 0.2 per 100 000 in the aged 0‐9 group, and from 0.2 to 0.3 per 100 000 in the aged 20‐29 group, respectively, over three decades. The incidence was fairly consistent with 0.4 per 100 000 no matter in poor or affluent areas over the three decades. In addition, Whites and Blacks shared the same incidence with 0.4 per 100 000 in the three decades (Figure 1, Table[Link]). In subgroups of all patients aged 0‐29 years, the incidence was 0.4, 0.5, and 0.4 per 100 000, respectively, for males, and 0.3, 0.3, and 0.4 per 100 000, respectively, for females in the three decades. However, the incidence was significantly higher in males than females in the aged 10‐19 group, despite the difference in incidence between sexes narrowing from 0.4 to 0.2 per 100 000 in the last two decades (Figure S1).

### 3.2 Relative survival tendency in osteosarcoma

**Figure 2:**
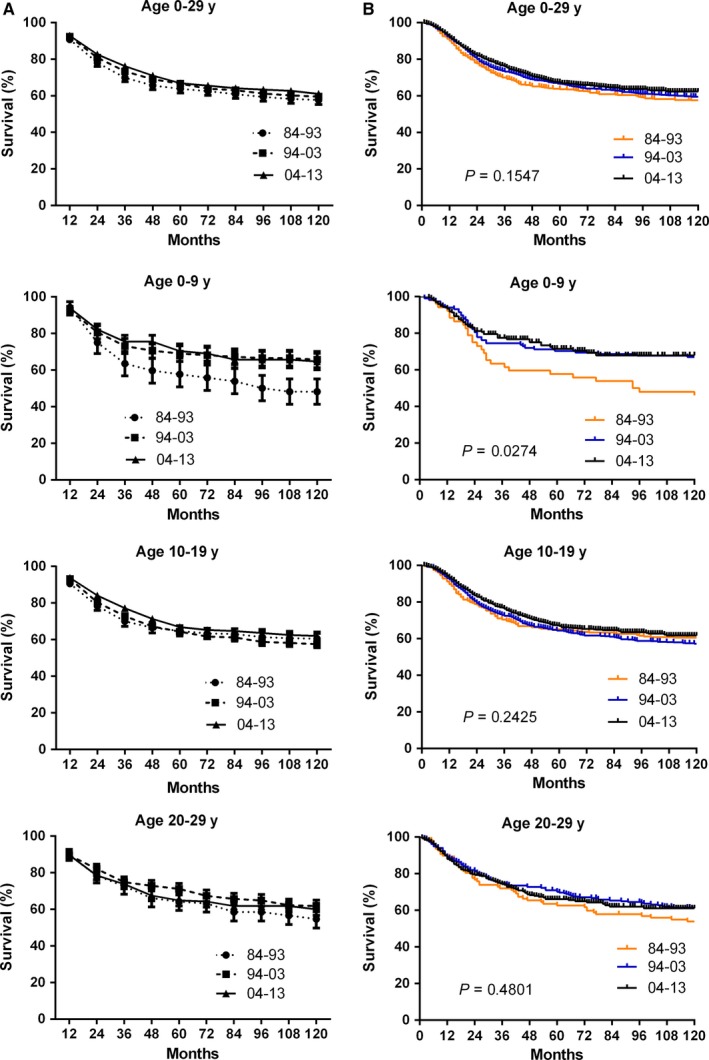
Trends in 10‐year relative survival rates (A) and Kaplan‐Meier survival analyses (B) for patients with osteosarcoma at 18 SEER sites from 1984 to 1993 (orange), 1994‐2003 (blue), and 2004‐2013 (black) according to age group (total and age 0‐9, 10‐19, and 20‐29 years) and calendar period

Total 2925 patients with osteosarcoma were identified during 1984‐2013 at 18 original SEER sites. There was an improvement in the relative survival rate (RSR) in aged 0‐29 years over three decades, with 10‐year RSR increasing from 57.7% to 59.5% to 61.0% in each respective decade. The increasing tendency was observed in all age groups over three decades, especially for the aged 0‐9 group with the greatest increase from 48.2% to 64.7% (Figure 2A). The Kaplan‐Meier survival analysis also demonstrated the significantly improved survival in aged 0‐9 group in the three decades with *P* value = 0.0274 (Figure 2B). In addition, the 10‐year RSR increased from 60.5% to 62.0% in the aged 10‐19 group, and from 54.7% to 60.0% in the aged 20‐29 group, respectively, in the three decades (Table 1).

**Table 1:** Relative survival rates of osteosarcoma patients during the periods of 1984‐1993, 1994‐2003, and 2004‐2013 at 18 SEER sites

Age group	Decade		
	1984‐1993	1994‐2003	2004‐2013
12‐mo RS			
0‐29	90.8 ± 1.4 (445)	92.3 ± 0.9 (991)	92.9 ± 0.7 (1489)
0‐9	94.2 ± 3.2 (52)	92.5 ± 2.4 (119)	93.9 ± 1.8 (195)
10‐19	90.6 ± 1.7 (287)	93.2 ± 1.0 (633)	93.8 ± 0.8 (956)
20‐29	89.7 ± 4.0 (106)	89.6 ± 2.0 (239)	89.7 ± 1.7 (338)*
24‐mo RS			
0‐29	78.0 ± 2.0	80.9 ± 1.3	82.6 ± 1.1
0‐9	75.0 ± 6.0	80.7 ± 3.6	82.2 ± 3.0
10‐19	78.4 ± 2.4	80.4 ± 1.6	84.1 ± 1.3*
20‐29	78.4 ± 4.0	82.1 ± 2.5	78.5 ± 2.4
36‐mo RS			
0‐29	69.9 ± 2.2	73.4 ± 1.4	76.3 ± 1.2*
0‐9	63.5 ± 6.7	73.1 ± 4.1	75.5 ± 3.5
10‐19	70.0 ± 2.7	72.9 ± 1.8	77.2 ± 1.5*
20‐29	72.7 ± 4.4	74.9 ± 2.8	73.8 ± 2.6
48‐mo RS			
0‐29	65.6 ± 2.3	69.0 ± 1.5	71.0 ± 1.3*
0‐9	59.7 ± 6.8	70.6 ± 4.2	75.5 ± 3.5*
10‐19	66.5 ± 2.8	67.2 ± 1.9	71.4 ± 1.7
20‐29	66.1 ± 4.7	72.9 ± 2.9	67.5 ± 2.9
60‐mo RS			
0‐29	63.8 ± 2.3	66.6 ± 1.5	66.9 ± 1.4
0‐9	57.7 ± 6.9	69.0 ± 4.2	70.4 ± 3.9
10‐19	64.8 ± 2.8	64.4 ± 1.9	66.9 ± 1.8
20‐29	64.2 ± 4.7	71.2 ± 3.0	64.9 ± 3.0
72‐mo RS			
0‐29	62.5 ± 2.3	63.9 ± 1.5	65.5 ± 1.5
0‐9	55.8 ± 6.9	68.1 ± 4.3	69.0 ± 4.1
10‐19	63.4 ± 2.9	61.7 ± 1.9	65.3 ± 1.9
20‐29	63.2 ± 4.7	67.4 ± 3.1	64.3 ± 3.1
84‐mo RS			
0‐29	60.9 ± 2.3	63.0 ± 1.5	64.1 ± 1.6
0‐9	53.9 ± 6.9	67.3 ± 4.3	65.7 ± 4.5
10‐19	63.1 ± 2.9	61.2 ± 2.0	64.7 ± 1.9
20‐29	58.5 ± 4.9	65.7 ± 3.1	61.8 ± 3.3
96‐mo RS			
0‐29	59.4 ± 2.4	61.2 ± 1.6	63.4 ± 1.6
0‐9	50.1 ± 6.9	66.5 ± 4.3*	65.7 ± 4.5
10‐19	61.4 ± 2.9	58.8 ± 2.0	63.6 ± 2.0
20‐29	58.5 ± 4.9	64.9 ± 3.2	61.8 ± 3.3
108‐mo RS			
0‐29	58.3 ± 2.4	60.2 ± 1.6	62.7 ± 1.7
0‐9	48.2 ± 6.9	66.5 ± 4.3*	65.7 ± 4.5
10‐19	60.8 ± 2.9	58.2 ± 2.0	62.5 ± 2.1
20‐29	56.6 ± 4.9	62.3 ± 3.2	61.8 ± 3.3
120‐mo RS			
0‐29	57.7 ± 2.4	59.5 ± 1.6	61.0 ± 1.7
0‐9	48.2 ± 6.9	65.7 ± 4.4*	64.7 ± 4.5
10‐19	60.5 ± 2.9	57.5 ± 2.0	62.0 ± 2.1
20‐29	54.7 ± 4.9	61.8 ± 3.2	60.0 ± 3.3

Data are mean ± standard error of the mean, with number of patients in parentheses.

mo, month; RS, relative survival; SEM, standard error of the mean.

*P < 0.01 for comparisons with the preceding decade.

**Figure 3:**
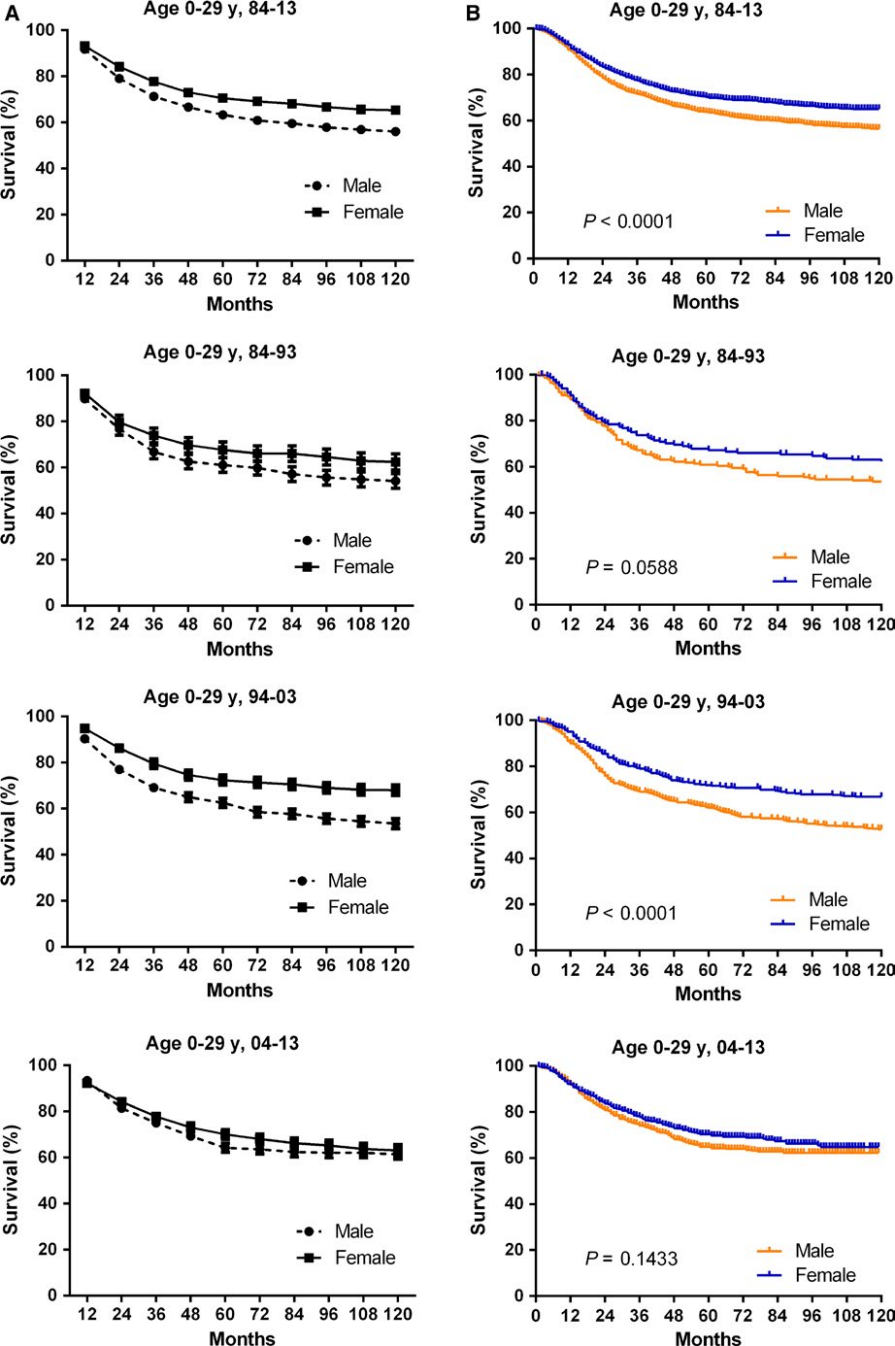
Trends in 10‐year relative survival rates and Kaplan‐Meier survival analyses according to sex (A, B) including Male (orange) and Female(blue) for patients with osteosarcoma from 18 SEER sites from 1984 to 2013. Data are shown by sex and calendar period over the three decades

The improved RSRs were observed in both sexes, with greater survival increase in males over three decades (Figures 3, S2). And the survival difference between sexes became narrowed from 8.3% to 1.5% in the three decades. The 10‐year RSRs improved from 54.1% to 61.5% in males, and from 62.4% to 63.0% in females, respectively, in the three decades (Table 2).

**Table 2:** Five‐year and 10‐year relative survival rates of osteosarcoma patients according to sex, age group, and calendar period from 1984 to 2013 at 18 SEER sites

Decade	Age group	Sex	
		Male	Female
84‐93	60‐mo RS		
	0‐29	61.0 ± 3.1 (253)	67.6 ± 3.4 (192)
	0‐9	52.1 ± 10.0 (25)	63.0 ± 9.3 (27)
	10‐19	63.4 ± 3.8 (163)	66.7 ± 4.3 (124)
	20‐29	58.3 ± 6.2 (65)	73.1 ± 7.0 (65)
	120‐mo RS		
	0‐29	54.1 ± 3.2	62.4 ± 3.5
	0‐9	40.1 ± 9.8	55.6 ± 9.6
	10‐19	58.8 ± 3.9	62.7 ± 4.4
	20‐29	47.7 ± 6.3	65.6 ± 7.6
94‐03	60‐mo RS		
	0‐29	62.5 ± 2.0 (581)	72.4 ± 2.2 (410)**
	0‐9	67.3 ± 6.0 (61)	70.7 ± 6.0 (58)
	10‐19	59.9 ± 2.5 (385)	71.4 ± 2.9 (248)**
	20‐29	67.7 ± 4.1 (135)	75.7 ± 4.3 (104)
	120‐mo RS		
	0‐29	53.5 ± 2.1	68.0 ± 2.3***
	0‐9	62.5 ± 6.2	69.0 ± 6.1
	10‐19	51.3 ± 2.6	67.0 ± 3.0***
	20‐29	55.8 ± 4.4	69.7 ± 4.6*
04‐13	60‐mo RS		
	0‐29	64.2 ± 2.0 (812)	70.0 ± 2.1 (677)*
	0‐9	69.8 ± 5.7 (96)	71.5 ± 5.3 (99)
	10‐19	65.0 ± 2.5 (537)	69.3 ± 2.6 (419)
	20‐29	59.4 ± 4.3 (179)	71.4 ± 4.2 (159)*
	120‐mo RS		
	0‐29	61.5 ± 2.1	63.0 ± 2.5
	0‐9	62.0 ± 6.9	68.0 ± 5.8
	10‐19	61.5 ± 2.7	62.3 ± 3.2
	20‐29	59.0 ± 4.3	63.4 ± 5.1

Data are means ± standard error of the mean, with number of patients in parentheses.

Mo, month; RSR, relative survival rate; SEM, standard error of the mean.

*P <* *0.01, **P <* *0.001, and ***P <* *0.0001 for comparisons with the Male group.

In addition, age, sex, race, and SES were assessed by Cox regression analyses after patients being divided by age. Sex served as an independent risk factor with *P *<* *0.001 in total patients aged 0‐29 years. Females, with a hazard ratio (HR) of 0.741, was associated with a lower risk of death in osteosarcoma compared to males (Table 3).

**Table 3:** Summary data for Cox regression analysis of survival in patients aged 0‐29 years with osteosarcoma from 1984 to 2013 at 18 SEER sites

Variable	Univariate			Multivariate		
	95% CI	HR	*P*	95% CI	HR	*P*
Age (y)						
N		1.0			1.0	
N + 1	1.001‐1.023	1.011	0.043	0.998‐1.021	1.010	0.076
Sex						
Male		1.0			1.0	
Female	0.645‐0.843	0.737	<0.001	0.645‐0.843	0.741	<0.001
Race						
White		1.0				
Black	0.879‐1.241	1.045	0.618			
Other	0.901‐1.111	1.001	0.990			
SES						
Low‐poverty		1.0				
Medium‐poverty	0.921‐1.196	1.049	0.472			
High‐poverty	0.991‐1.256	1.115	0.072			

95% CI, 95% confidence interval; HR, hazard risk; SES, socioeconomic status. Age is a continuous variable. N represents age.

**Figure 4:**
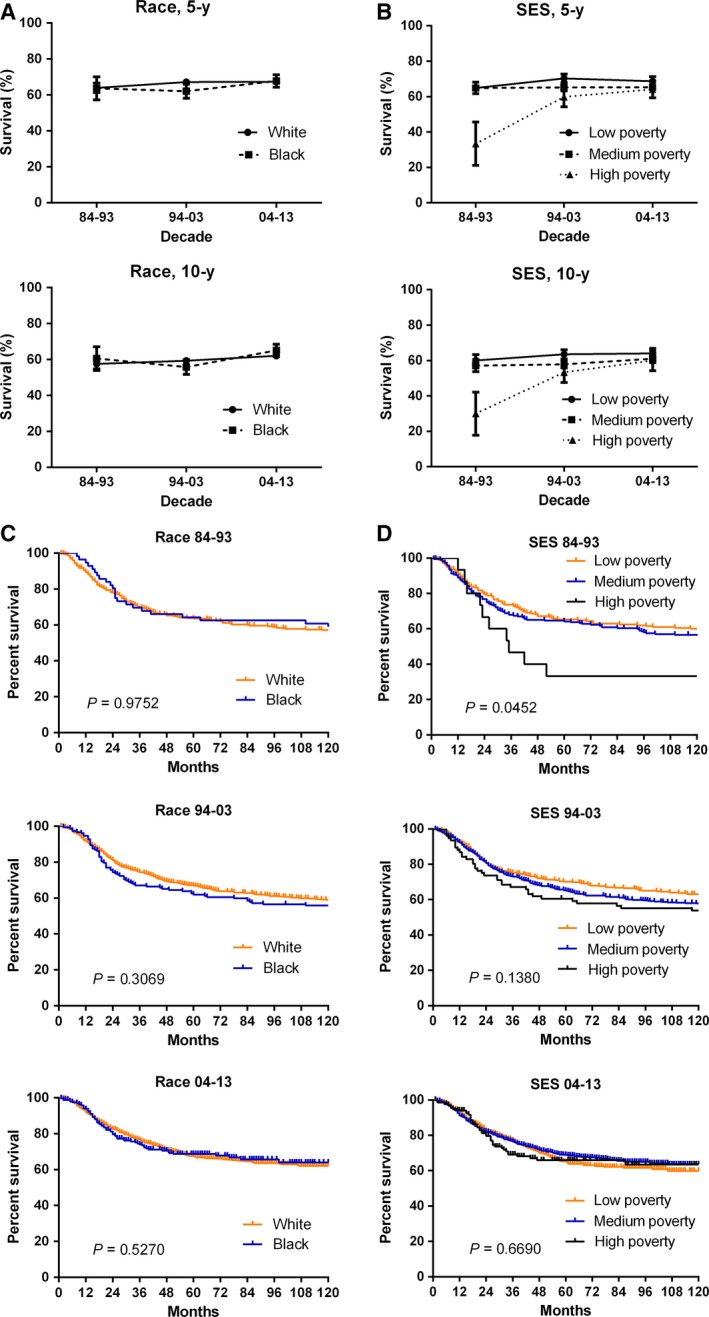
Five‐year and 10‐year relative survival rates and Kaplan‐Meier survival analyses according to race (A, C) including White (orange) and Black (blue), and SES/county‐level poverty rates (B, D) including Low‐poverty (orange), Medium‐poverty (blue) and High‐poverty (black), for patients with osteosarcoma at 18 SEER sites from 1984 to 2013

The survival improved for Whites and Blacks over the three decades, but there was no significantly survival difference between Whites and Blacks in 10‐year survival for Kaplan‐Meier survival analysis (Figure 4C). The 10‐year RSRs for Whites and Blacks, respectively, improved from 57.7% and 56.0 in the first decade to 62.0% and 65.0% in the last decade (Figure 4A, Table S2).

It was also observed that the survival improved in all SES subgroups, with the survival gap between the low‐ and high‐poverty groups significantly narrowing from 30% to 4% in the three decades (Figure 4B). The 10‐year RSR increased from 60.0% to 64.0% in low‐poverty group, and from 57.1% to 61% in medium‐poverty group, respectively, furthermore, it dramatically increased in the high‐poverty group, from 30.0% to 60.0% over three decades (Table S3). Kaplan‐Meier survival analysis indicated the survival significantly differed among three SES subgroups in the first decade only (*P *=* *0.0452), not in the last two decades (*P *=* *0.138 and *P *=* *0.669, respectively, Figure 4D).

## 4 DISCUSSION

Our study demonstrated that the overall osteosarcoma incidence of aged 0‐29 years appeared to be relatively stable with the peak incidence occurring in the aged 10‐19 group over the three decades. The 10‐year relative survival improved in all age groups, especially for the aged 0‐9 group with the greatest increase from 48.2% to 65.7% in the three decades. Additionally, the improved survival was greater in males than in females, and the survival gap between sexes was narrowing over time in the three decades. Furthermore, there was a significant progressive relative survival increase from 30% to 60% in high‐poverty patients, with the survival gap between low‐ and high‐poverty groups significantly narrowing over time in the three decades.

We observed that the overall incidence of osteosarcoma appeared stable, and whether Whites or Blacks, poor or affluent groups, the incidence was fairly consistent with 0.4 per 100000 over the three decades. The result may be attributed to variables or the fact that the etiology of osteosarcoma had little relation with the different races or SES in the U.S.

In addition, our data showed that the incidence difference between sexes became narrowed over time in the three decades. It was originally reported that males were affected with osteosarcoma more frequently than females.^1^, ^15^, ^16^, ^17^ However, recent reports suggested that the osteosarcoma incidence may be equal in both sexes.^18^, ^19^ This finding was consistent with our study that the males shared the similar incidence with the females in the last decade. Additionally, the peak incidence occurred in the aged 10‐19 years and reached higher in males compared to females, which suggested that bone growth and/or hormonal changes during puberty may play an important role in osteosarcoma etiology.^20^, ^21^ This relationship between osteosarcoma, bone growth, and hormones may also partly explain the slightly higher overall incidence in males compared to females.

The relative survival improved in the all age groups, especially for the aged 0‐9 group in the three decades. The improvement in survival was closely related to rapid disease recognition and advances in applying surgery, radiotherapy, multi‐drug, multi‐cycle adjuvant chemotherapy regimes.^11^, ^22^ However, there was little progress in improving the survival of osteosarcoma in the three decades despite the multi‐modal approach, and treatment is accompanied by many early and late side effects.^23^, ^24^ This highlights the urgency of developing novel and more specific therapeutic approaches to improve the outcome of osteosarcoma.

Furthermore, the improved survival for males was greater than for females despite the fact that survival advantage was still observed in females compared to males. The true cause of the survival difference between sexes is unclear, but the observation suggested that the biological or genetic disparities may play an important role in response of osteosarcoma treatment.^25^, ^26^

In subgroups of race, the improved RSR was found in both White and Blacks, but no significant survival difference was observed between Whites and Blacks over time in the three decades, which may be attributed to variables or the fact that all patients with different races could receive the indiscriminate treatment. In terms of SES subgroups, the survival significantly increased in the high‐poverty group, and the gap among SES subgroups kept narrowing over time in the three decades. SES, including household income, insurance status, education, and poverty, is increasing critical for disease prognosis with timely and more effective treatment resources in several cancers.^27^, ^28^ Compared to patients with low poverty, high‐poverty patients were more likely to reside in counties in which usually were lower health insurance coverage, diminished access to health care providers and fewer oncologist, all of which were related to a delay in diagnosis and higher risk of early death. Indeed, a delay in diagnosis substantially increases the risk of an advanced stage of disease at presentation, which is significantly associated with decreased survival.^29^, ^30^, ^31^, ^32^ However, with the increasing improvement of health care policies and medical insurance, the patients with low income could also have access to medical consultation and better treatment resources, thereby they could have a progress survival over time.^33^, ^34^

For the Cox regression analysis, SES did not appear as an independent prognostic factor despite the fact that there was a significant improvement in relative survival in the high‐poverty group, which may be, at least partially, attributed to small number of 226 cases in high‐poverty group.

To our knowledge, Surveillance, Epidemiology, and End Results program is the largest database of the incidence and survival of osteosarcoma in the U.S. so far. But it is noteworthy that there still are several limitations of the study. Firstly, the data about incidence and survival reflect only specific areas that provided the data over the past three decades. Secondly, the study may be affect by error and bias, such as under‐registration or misclassification of cases and variation of socioeconomic status within and among counties may bias estimates.^12^, ^35^ Thirdly, it was not analyzed based on stage and did not have the details of treatment in the osteosarcoma patients in this study, which may have differed in relative survival.

Overall, the osteosarcoma incidence of aged 0‐29 years kept relatively stable, with an improvement in relative survival over the three decades. Although survival advantage was still observed in females, the improved survival for males was greater than for females, and the survival gap between sexes became narrowed in the three decades. Furthermore, the survival dramatically increased in high‐poverty group, indicating that patients with low income can also have access to better medical consultation and receiving effective and consistent treatment without distinction, as the result of increasing improved the health care system.

## CONFLICT OF INTEREST

The authors declare no conflict of interest.
