# Supplementary material for: Increased survival of patients aged 0‐29 years with osteosarcoma: A period analysis, 1984‐2013
Source: Cancer Med. 2018 Jul 10;7(8):3652–61. doi: 10.1002/cam4.1659 (PMC6089162; doi:10.1002/cam4.1659)
Supplement: Supplementary file 3 [file CAM4-7-3652-s003.docx]

**Increased survival of patients aged 0-29 years with osteosarcoma: a period analysis, 1984-2013.**

Jinna Wu^1,*^, Huanhuan Sun^1,*^, Jie Li^2^, Yuanqing Guo^3^, Kuibo Zhang^3^, Chuandong Lang^4^, Changye Zou^5^ and Haiqing Ma MD, PhD^1^

^1^ Department of Oncology, The Fifth Affiliated Hospital of Sun Yat-sen University, Zhuhai, Guangdong 519000, China

^2^ Department of Breast and Thyroid Surgery, The First Affiliated Hospital of Sun Yat-Sen University, Guangzhou, Guangdong 510080, China

^3^ Department of Spinal Surgery, The Fifth Affiliated Hospital of Sun Yat-sen University, Zhuhai, Guangdong 519000, China

^4^ Department of Orthopedic, The First Affiliated Hospital of Sun Yat-Sen University, Guangzhou, Guangdong 510080, China

^5^ Department of Orthopedic Oncology, The First Affiliated Hospital of Sun Yat-Sen University, Guangzhou, Guangdong 510080, China

*These authors have contributed equally to this work.

Correspondence to: Haiqing Ma, Department of Oncology, The Fifth Affiliated Hospital of Sun Yat-sen University, 52 Meihua Dong Rd., Zhuhai, Guangdong, China +86-0756-2528888, email: mahaiqing@mail.sysu.edu.cn and Changye Zou, email: zhouchangye1@163.com

**Supplementary Table S1**.The incidence of osteosarcoma according to age group and decade within sex, race and SES groups from 1984 to 2013 at the nine original SEER sites. Data are incidence per 100,000 people by year of diagnosis, with the number of patients in parentheses.

|  | |  | **Age** | **Decade** | | | |
| --- | --- | --- | --- | --- | --- | --- | --- |
| **Variable** | |  | **Group** | | **1984-1993** | **1994-2003** | **2004-2013** |
| Total |  | |  | |  |  |  |
|  |  | | 0-29 | | 0.4 (397) | 0.4 (457) | 0.4 (460) |
|  |  | | 0-9 | | 0.1 (44) | 0.1 (53) | 0.2 (60) |
|  |  | | 10-19 | | 0.8 (259) | 0.8 (301) | 0.7 (283) |
|  |  | | 20-29 | | 0.2 (94) | 0.3 (103) | 0.3 (117) |
| Sex | Male | |  | |  |  |  |
|  |  | | 0-29 | | 0.4 (227) | 0.5 (227) | 0.4 (256) |
|  |  | | 0-9 | | 0.1 (22) | 0.1 (24) | 0.2 (31) |
|  |  | | 10-19 | | 0.9 (147) | 1.0 (194) | 0.8 (166) |
|  |  | | 20-29 | | 0.3 (58) | 0.3 (59) | 0.3 (59) |
|  | Female | |  | |  |  |  |
|  |  | | 0-29 | | 0.3 (170) | 0.3 (180) | 0.4 (204) |
|  |  | | 0-9 | | 0.1 (22) | 0.2 (29) | 0.2 (29) |
|  |  | | 10-19 | | 0.7 (112) | 0.6 (107) | 0.6 (117) |
|  |  | | 20-29 | | 0.2 (36) | 0.2 (44) | 0.3 (58) |
| Race | White | |  | |  |  |  |
|  |  | | 0-29 | | 0.4 (301) | 0.4 (329) | 0.4 (336) |
|  |  | | 0-9 | | 0.1 (33) | 0.1 (36) | 0.2 (44) |
|  |  | | 10-19 | | 0.8 (194) | 0.8 (213) | 0.7 (204) |
|  |  | | 20-29 | | 0.2 (74) | 0.3 (80) | 0.3 (88) |
|  | Black | |  | |  |  |  |
|  |  | | 0-29 | | 0.4 (53) | 0.4 (68) | 0.4 (76) |
|  |  | | 0-9 | | 0.1 (6) | 0.2 (11) | 0.1 (8) |
|  |  | | 10-19 | | 0.8 (35) | 0.9 (46) | 0.8 (51) |
|  |  | | 20-29 | | 0.3 (12) | 0.2 (11) | 0.3 (17) |
|  | Other | |  | |  |  |  |
|  |  | | 0-29 | | 0.5 (43) | 0.5 (60) | 0.3 (48) |
|  |  | | 0-9 | | 0.2 (5) | 0.1 (6) | 0.2 (8) |
|  |  | | 10-19 | | 1.0 (30) | 1.0 (42) | 0.5 (28) |
|  |  | | 20-29 | | 0.2 (8) | 0.3 (12) | 0.2 (12) |
| SES | Low Poverty | | 0-29 | | 0.4 (207) | 0.4 (259) | 0.4 (260) |
|  |  | | 0-9 | | 0.1 (24) | 0.2 (32) | 0.2 (37) |
|  |  | | 10-19 | | 0.8 (132) | 0.8 (163) | 0.7 (160) |
|  |  | | 20-29 | | 0.3 (51) | 0.3 (64) | 0.3 (63) |
|  | Med-high Poverty | | 0-29 | | 0.4 (190) | 0.4 (197) | 0.4 (200) |
|  |  | | 0-9 | | 0.1 (20) | 0.1 (21) | 0.2 (23) |
|  |  | | 10-19 | | 0.8 (127) | 0.8 (137) | 0.8 (123) |
|  |  | | 20-29 | | 0.2 (43) | 0.2 (39) | 0.3 (54) |

**Supplementary Table S2**. 5-year and 10-year relative survival rates of osteosarcoma patients according to race, age group, and calendar period from 1984 to 2013 at 18 SEER sites. Data are means ± standard error of the mean, with number of patients in parentheses.

|  | **Age** | | **Race** | | | |
| --- | --- | --- | --- | --- | --- | --- |
| **Decade** | | **Group** | | **White** | **Black** | **Other** |
| 84-93 | | 60-Mo RS | |  |  |  |
|  | | 0-29 | | 63.9 ± 2.6 (338) | 63.6 ± 6.4 (57) | 63.9 ± 6.8 (50) |
|  | | 0-9 | | 55.3 ± 8.1 (38) | 57.2 ± 18.7 (7) | 71.5 ± 17.1 (7) |
|  | | 10-19 | | 65.4 ± 3.3 (217) | 63.5 ± 7.9 (38) | 62.2 ± 8.7 (32) |
|  | | 20-29 | | 63.7 ± 5.4 (83) | 67.2 ± 13.7 (12) | 64.7 ± 14.5 (11) |
|  | | 120-Mo RS | |  |  |  |
|  | | 0-29 | | 57.7 ± 2.7 | 56.0 ± 6.6 | 55.8 ± 7.1 |
|  | | 0-9 | | 44.8 ± 8.1 | 57.2 ± 18.7 | 57.2 ± 18.7 |
|  | | 10-19 | | 61.4 ± 3.3 | 58.7± 8.1 | 55.8 ± 8.9 |
|  | | 20-29 | | 52.6 ± 5.6 | 67.2 ± 13.7 | 54.8 ± 15.1 |
| 94-03 | | 60-Mo RS | |  |  |  |
|  | | 0-29 | | 67.1 ± 1.8 (729) | 64.0 ± 3.9 (154) | 69.4 ± 4.5 (108) |
|  | | 0-9 | | 68.3 ± 5.1 (82) | 65.3 ± 9.9 (23) | 78.6 ± 11.0 (14) |
|  | | 10-19 | | 64.9 ± 2.3 (452) | 59.5 ± 4.9 (103) | 68.1 ± 5.3 (78) |
|  | | 20-29 | | 71.9 ± 3.3 (195) | 68.3 ± 8.9 (28) | 67.4 ± 12.1 (16) |
|  | | 120-Mo RS | |  |  |  |
|  | | 0-29 | | 59.3 ± 1.8 | 57.5 ± 4.1 | 65.8 ± 4.6 |
|  | | 0-9 | | 64.7 ± 5.3 | 65.3 ± 9.9 | 71.5 ± 12.1 |
|  | | 10-19 | | 57.5 ± 2.4 | 52.0 ± 5.0 | 64.4 ± 5.5 |
|  | | 20-29 | | 61.3 ± 3.6 | 61.7 ± 9.4 | 67.4 ± 12.1 |
| 04-13 | | 60-Mo RS | |  |  |  |
|  | | 0-29 | | 67.3 ± 1.6 (1130) | 67.7 ± 3.5 (240) | 61.3 ± 5.4 (119) |
|  | | 0-9 | | 70.7 ± 4.7 (138) | 72.6 ± 8.6 (37) | 65.3 ± 11.6 (20) |
|  | | 10-19 | | 66.9 ± 2.1 (732) | 67.7 ± 4.4 (158) | 65.2 ± 7.2 (66) |
|  | | 20-29 | | 66.6 ± 3.4 (260) | 64.8 ± 8.1 (45) | 51.8 ± 10.8 (33) |
|  | | 120-Mo RS | |  |  |  |
|  | | 0-29 | | 62.0 ± 1.9 | 65.0 ± 3.5 | 55.0 ± 5.8 |
|  | | 0-9 | | 65.7 ± 4.5 | 72.0 ± 8.6 | 56.0 ± 13.2 |
|  | | 10-19 | | 61.0 ± 2.5 | 67.0 ± 4.4 | 58.7 ± 7.8 |
|  | | 20-29 | | 62.8 ± 3.7 | 64.8 ± 8.1 | 51.8 ± 10.8 |

Abbreviations: Mo, month; RS, relative survival; SEM, standard error of the mean.

**p* < 0.01, ***p* < 0.001, and ****p* < 0.0001 for comparisons with the White group.

**Supplementary Table S3**. 5-year and 10-year relative survival rates of osteosarcoma patients according to SES, age group, and calendar period from 1984 to 2013 at 18 SEER sites. Data are means ± standard error of the mean, with number of patients in parentheses.

|  | **Age** | | **SES** | | | |
| --- | --- | --- | --- | --- | --- | --- |
| **Decade** | | **Group** | | **Low Poverty Medium Poverty High Poverty** | | |
| 84-93 | | 60-Mo RS | |  |  |  |
|  | | 0-29 | | 64.9 ± 3.3 (213) | 64.9 ± 3.3 (217) | 33.4 ± 12.2 (15)* |
|  | | 0-9 | | 50.0 ± 10.2 (24) | 66.7 ± 9.1 (27) | 0.0 ± 0.0 (1) |
|  | | 10-19 | | 66.2 ± 4.1 (135) | 65.2 ± 4.0 (142) | 40.1 ± 15.5 (10) |
|  | | 20-29 | | 68.4 ± 6.4 (54) | 62.8 ± 7.0 (48) | 25.1 ± 21.7 (4) |
|  | | 120-Mo RS | |  |  |  |
|  | | 0-29 | | 60.0 ± 3.4 | 57.1 ± 3.4 | 30.0 ± 12.2* |
|  | | 0-9 | | 45.9 ± 10.2 | 52.0 ± 9.6 | 0.0 ± 0.0 |
|  | | 10-19 | | 61.9 ± 4.2 | 60.5 ± 4.2 | 40.1 ± 15.5 |
|  | | 20-29 | | 61.2 ± 6.8 | 49.8 ± 7.4 | 25.1 ± 21.7 |
| 94-03 | | 60-Mo RS | |  |  |  |
|  | | 0-29 | | 70.3 ± 2.4 (357) | 65.2 ± 2.0 (555) | 59.9 ± 5.6 (78) |
|  | | 0-9 | | 72.8 ± 6.7 (44) | 65.7 ± 5.9 (64) | 72.8 ± 13.4 (11) |
|  | | 10-19 | | 66.0 ± 3.2 (224) | 63.9 ± 2.6 (358) | 62.2 ± 6.9 (50) |
|  | | 20-29 | | 80.0 ± 4.3 (89) | 68.6 ± 4.1 (133) | 43.9 ± 12.4 (17)** |
|  | | 120-Mo RS | |  |  |  |
|  | | 0-29 | | 63.5 ± 2.6 | 57.9 ± 2.1 | 53.4 ± 5.8 |
|  | | 0-9 | | 68.8 ± 6.7 | 61.0 ± 6.1 | 60.7 ± 15.8 |
|  | | 10-19 | | 59.0 ± 3.3 | 56.5 ± 2.6 | 58.3 ± 7.0 |
|  | | 20-29 | | 69.9 ± 5.0 | 60.2 ± 4.3 | 29.4 ± 11.9** |
| 04-13 | | 60-Mo RS | |  |  |  |
|  | | 0-29 | | 68.7 ± 2.6 (489) | 65.3 ± 1.8 (867) | 64.1 ± 4.8 (133) |
|  | | 0-9 | | 72.5 ± 6.6 (66) | 72.3 ± 5.0 (114) | 41.2 ± 17.1 (15) |
|  | | 10-19 | | 64.5 ± 3.2 (320) | 68.6 ± 2.3 (543) | 65.6 ± 5.8 (93) |
|  | | 20-29 | | 58.3 ± 5.7 (103) | 67.3 ± 3.7 (210) | 75.9 ± 9.5 (25) |
|  | | 120-Mo RS | |  |  |  |
|  | | 0-29 | | 64.0 ± 2.9 | 61.0 ± 2.1 | 60.0 ± 5.8 |
|  | | 0-9 | | 67.0 ± 7.6 | 67.1 ± 5.8 | 35.5 ± 17.1 |
|  | | 10-19 | | 59.0 ± 3.9 | 64.0 ± 2.7 | 60.0 ± 7.0 |
|  | | 20-29 | | 56.0 ± 5.9 | 63.0 ± 4.2 | 70.5 ± 9.5 |

Abbreviations: Mo, month; RS, relative survival; SEM, standard error of the mean.

**p* < 0.01, ***p* < 0.001, and ****p* < 0.0001 for comparisons with the White group.
